# Updated perspectives on the contribution of the microbiome to the pathogenesis of mucositis using the MASCC/ISOO framework

**DOI:** 10.1007/s00520-024-08752-4

**Published:** 2024-07-31

**Authors:** Andrea M. Stringer, Benjamin M. Hargreaves, Rui Amaral Mendes, Nicole M. A. Blijlevens, Julia S. Bruno, Paul Joyce, Srinivas Kamath, Alexa M. G. A. Laheij, Giulia Ottaviani, Kate R. Secombe, Arghavan Tonkaboni, Yehuda Zadik, Paolo Bossi, Hannah R. Wardill

**Affiliations:** 1 Clinical and Health Sciences, University of South Australia, Adelaide, 5000 Australia; 2 Faculty of Medicine, University of Porto/CINTESIS@RISE, Porto, Portugal; 3 Present Address: Department of Oral and Maxillofacial Medicine and Diagnostic Sciences, Case Western Reserve University, Cleveland, OH 44106-7401 USA; 4 Department of Hematology, Radboud University Medical Center, Nijmegen, The Netherlands; 5 Molecular Oncology Center, Hospital Sírio-Libanês, São Paulo, Brazil; 6 Centre for Pharmaceutical Innovation, Clinical and Health Sciences, University of South Australia, Adelaide, 5000 Australia; 7 Department of Oral Medicine, Academic Centre for Dentistry Amsterdam, University of Amsterdam and VU University, Amsterdam, The Netherlands; 8 Present Address: Department of Oral and Maxillofacial Surgery, UMC, University of Amsterdam, Amsterdam, The Netherlands; 9 Department of Surgical, Medical and Health Sciences, University of Trieste, Trieste, Italy; 10 The School of Biomedicine, Faculty of Health and Medical Sciences, The University of Adelaide, Adelaide, 5005 Australia; 11 Department of Oral Medicine, School of Dentistry, Tehran University of Medical Sciences, Tehran, Iran; 12 Department of Military Medicine and “Tzameret”, Faculty of Medicine, The Hebrew University of Jerusalem, Jerusalem, Israel; 13 Department of Oral Medicine, Sedation and Imaging, Faculty of Dental Medicine, The Hebrew University of Jerusalem, Jerusalem, Israel; 14 Department of Biomedical Sciences, Humanitas University, Via Rita Levi Montalcini 4, Pieve Emanuele, 20072 Milan, Italy; 15 IRCCS Humanitas Research Hospital, Via Manzoni 56, Rozzano, 20089 Milan, Italy; 16 Supportive Oncology Research Group, Precision Cancer Medicine Theme, South Australian Health and Medical Research Institute, Level 5S, Adelaide, 5000 Australia

**Keywords:** Mucositis, Gut microbiota, Oral microbiome, Dysbiosis, Cancer therapy

## Abstract

Advances in the treatment of cancer have significantly improved mortality rates; however, this has come at a cost, with many treatments still limited by their toxic side effects. Mucositis in both the mouth and gastrointestinal tract is common following many anti-cancer agents, manifesting as ulcerative lesions and associated symptoms throughout the alimentary tract. The pathogenesis of mucositis was first defined in 2004 by Sonis, and almost 20 years on, the model continues to be updated reflecting ongoing research initiatives and more sophisticated analytical techniques. The most recent update, published by the Multinational Association for Supportive Care in Cancer and the International Society for Oral Oncology (MASCC/ISOO), highlights the numerous co-occurring events that underpin mucositis development. Most notably, a role for the ecosystem of microorganisms that reside throughout the alimentary tract (the oral and gut microbiota) was explored, building on initial concepts proposed by Sonis. However, many questions remain regarding the true *causal* contribution of the microbiota and associated metabolome. This review aims to provide an overview of this rapidly evolving area, synthesizing current evidence on the microbiota’s contribution to mucositis development and progression, highlighting (i) components of the 5-phase model where the microbiome may be involved, (ii) methodological challenges that have hindered advances in this area, and (iii) opportunities for intervention.

## Introduction

Although there have been major advances in the treatment of cancer, namely the development of novel treatment modalities, cytotoxic chemotherapy and radiotherapy remain the backbone of effective cancer control. Although effective, both chemotherapy and radiotherapy are highly non-selective, resulting in a range of healthy cells and tissues being subject to their cytotoxic properties resulting in tissue injury and the development of adverse symptoms and side effects. Mucosal tissues are especially susceptible to this non-selective cytotoxicity due to the highly proliferative nature of the mucosal epithelial cells. Damage to mucosal surfaces in the mouth and intestines (termed mucositis) is especially challenging, impacting oral intake, speech, swallowing, nutrient absorption, barrier integrity, and immune function [1]. It is also extremely prevalent, with oral mucositis (OM) occurring in almost 100% of people undergoing head and neck (chemo)radiotherapy, and gastrointestinal mucositis (GI-M) occurring in 80% of patients undergoing hematopoietic cell transplantation [2]. Due to the constellation of symptoms associated with mucositis, including pain, nausea, vomiting, diarrhea, weight loss, and infection, mucositis is a major clinical challenge, impacting treatment adherence, requiring costly resource utilization and impairing patient quality of life. Severe OM and GI-M can lead to treatment breaks or regimen de-escalation, impacting survival [3].

The core pathogenesis of mucositis is well established, initiated by direct cytotoxic damage to rapidly dividing stem cells [4]. While it is known that secondary inflammatory mechanisms serve to exacerbate this initial injury, the understanding of these mechanisms has continuously evolved, reflecting more sophisticated models of mucositis and investigational approaches [4]. The Multinational Association for Supportive Care in Cancer and the International Society for Oral Oncology (MASCC/ISOO) have systematically reviewed and updated the original 5-phase model of mucositis pathogenesis proposed by Sonis in 2004, with the most recent update published in 2019 [4]. In the 2019 update, the possible involvement of the oral and gut microbiota was discussed, along with tangible mechanisms for how these resident microorganisms may contribute to mucositis development causally. This marked a revolution in our understanding of host-microbe interactions in the context of mucositis development as, prior to this, microbes (including, but not limited to, bacteria, viruses, archaea, fungi, and yeast) had only been recognized for their opportunistic nature, in which they colonized areas of ulceration and potentially translocated into the blood stream. While this highlighted an important new component of mucositis pathogenesis, conclusions regarding the true *causal* role of the microbiota and its associated metabolome (“the microbiome” [5]) were limited due to the inherent challenges in defining causation with respect to the microbiome and, as such, a low number of studies that were appropriately designed to draw these conclusions. However, since the 2019 update, several studies have moved beyond these associative findings, and the contribution of the oral and gut microbiota to mucositis is increasingly evident. Here, we provide an updated perspective on the oral and gut microbiome contribution to mucositis pathogenesis while also placing a critical lens on the complex methodological approaches needed to dissect cause-and-effect.

## The host and its microbiome

The oral cavity and gastrointestinal tract (GIT), collectively termed the alimentary tract, are home to the human body’s richest and most complex microbial communities [6]. The human immune system and resident microorganisms have co-evolved over thousands of years, leading to a mutually beneficial relationship. Colonization of the mucosal surfaces early in life helps shape and mature the host’s immune system [7]. Despite a high load and diversity of microorganisms on several mucosal and skin surfaces, the immune system is tolerant of these commensal microorganisms, and this symbiotic alliance enables activation of protective immune responses towards pathogens and maintenance of immune tolerance responses to harmless (commensal) antigens [8, 9].

One of the key functions of the oral microbiome is to prevent colonization by external organisms, achieving this through competing for ligands, receptors, and nutrient substrates in the mouth. Furthermore, metabolic functions of oral microbiota dictate the oral microenvironment, creating a hostile environment that restricts the entry and expansion of exogenous organisms while also working to cross-feed commensals and thus maintain a healthy oral microbiota [10]. The oral microbiota can initiate chronic inflammation when the composition becomes dysbiotic (altered in its composition and diversity). Prominent disease examples arising from oral dysbiosis include periodontal-related diseases, *Helicobacter pylori* infection and gastric cancers [11, 12]. Overexpression of toll-like receptors (TLRs) is a key mechanism linking oral microbiota to disease pathogenesis, inducing nuclear factor kappa-light-chain-enhancer of activated B cells (NFκB) and downstream production of pro-inflammatory cytokines, such as interleukin (IL)-8 or tumor necrosis factor (TNF)-α [12–14].

Beyond the physical barrier that is established and maintained by the alimentary mucosa, pattern recognition receptors, such as TLRs, are the first line of immunological defense against pathogens [15, 16]. Located largely on the basolateral surface of epithelial cells, TLRs are well positioned to detect microbial products (e.g., molecular/danger/pathogen associated molecular patterns, MAMPs/DAMPs/PAMPs, respectively) that have breached the physical mucosal barrier [17]. Depending on the subtype, ligated TLRs will then initiate appropriate immune responses directed to either tolerate or eradicate the microbe [18, 19]. Typically, the eradication of pathogens is achieved by activating rapid innate immune responses characterized by the recruitment of neutrophils and the subsequent production of pro-inflammatory cytokines such as IL-1, IL-6, and TNF, as reviewed in detail here [16–19].

In addition to both microbial competition (i.e., colonization resistance), cross-feeding of other commensals, and direct interaction with TLRs, microorganisms in the gut are also capable of interacting with the host and its immune system via the production of metabolites, namely short-chain fatty acids (SCFAs) [20]. SCFAs are produced as a direct result of microbial-dependent fermentation of dietary fiber, with butyrate, acetate, and propionate the most abundant in the human GIT [21]. These metabolites (the microbial metabolome) are potent energy sources for intestinal enterocytes, responsible for tight junction assembly and integrity, mucus production, and enterocyte repair and replication [22]. With > 90% of these SCFAs reabsorbed in the colon, they also have the capacity to influence mucosal and systemic immune function, promoting regulatory T cells to elevate immune tolerance through the production of anti-inflammatory products, such as IL-10 [23]. As a result, when the gut microbiota is disrupted in a manner that reduces SCFAs, intestinal barrier function, mucosal repair/recovery, and immune function are all impaired. A loss of SCFAs also exacerbates microbial disruption, with SCFAs cross-feeding specific commensals and acidifying the luminal environment to restrict pathogen growth [20].

## Moving from opportunistic bystander to causal player—integrating the microbiome into the 5-phase model

### The 5-phase model of mucositis, from initiation to healing

The 5-phase mucositis model was first developed in 2004 by Stephen Sonis, highlighting a landmark moment in understanding this complex and highly burdensome toxicity [4]. In contrast to historical perspectives, this model highlighted the complex biological processes that underpin mucositis development, involving a dynamic interactive sequence of *pan-mucosal* events that not only initiate but also drive mucosal injury and its clinical sequelae [4]. In recognizing that mucositis involves more than just cytotoxic injury to the alimentary epithelium, Pandora’s box was opened, revealing a plethora of indirect inflammatory mechanisms that dictate both the depth and duration of mucositis. While the loss of mucosal integrity and the development of ulcers/lesions prone to superficial bacterial colonization is discussed, the mechanistic involvement of the oral and gut microbiota was limited and primarily inferred based on the interpretative of minimal research data available in the field [4].

Almost 20 years after the publication of the first 5-phase model, the role of the microbiome in mucositis development is increasingly clear, reflecting major advances in our ability to efficiently sequence the microbiome and the development of experimental models that enable precise augmentation of the microbiome. As a result, there is now a growing body of evidence that causally implicates the microbiome across all 5 phases of the mucositis model rather than only acknowledging their opportunistic involvement in Phase IV ulceration.

### Phase I: Initiation

The initiation phase involves *direct* damage to cells in the alimentary tract (mouth through to anus) caused by chemotherapy and radiotherapy treatments targeting these rapidly dividing populations of cells via irreversible damage to DNA [4]. This damage leads to an upregulation of inflammatory cytokine genes, targeting the basal epithelium and submucosa of the alimentary tract, leading to severe tissue damage.

The initiation of mucositis in both the oral cavity and GIT is almost exclusively driven by cytotoxic injury to stem cells, which are then incapable of populating the mucosa. Superficially, it may be challenging to consider how the microbiota may influence this relatively simple mechanism. The initial injury leads to the generation of oxidative stress through the production of reactive oxygen species (ROS). While antioxidants have been investigated for their ability to reduce chemotherapy and radiotherapy-induced toxicities (including mucosal), there is no consensus on the efficacy of antioxidants in OM or GI-M [3, 24, 25]. However, recent advances in the role of gut microbiota in intestinal disease generation suggest that ROS are increased during gut dysbiosis, and a healthy gut microbiota protects against oxidative stress [26], suggesting the state of the gut microbiota is likely to influence the onset (or not) of OM or GI-M. However, no studies have examined the effects of gut microbiota on ROS levels in mucositis (or ROS levels on microbiota). Therefore, further evidence is required to substantially link gut microbiota influences over ROS specifically to mucositis.

**Fig. 1:**
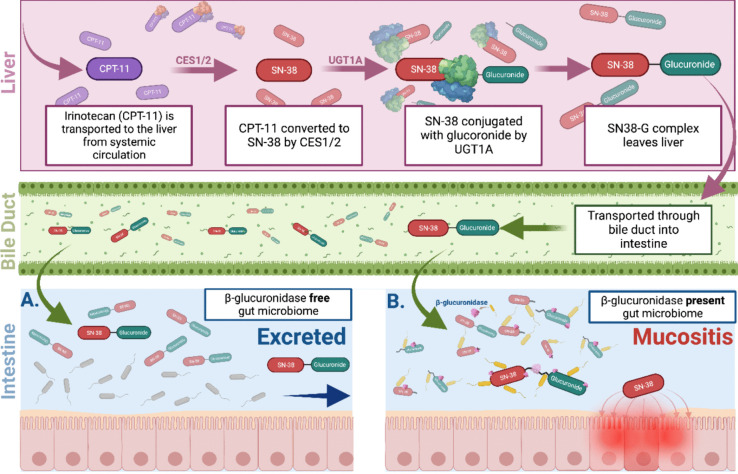
The severity of irinotecan-induced gastrointestinal mucositis is modulated by the gut microbiota through reactivation of SN38 by β-glucuronidase. Irinotecan (CPT-11) undergoes first-pass hepatic metabolism by carboxylesterase enzymes in the liver to form the active cytotoxic metabolite SN38. SN38 is conjugated to a glucuronide molecule to form the secondary non-toxic metabolite, SN38G, which is excreted into the GIT lumen via the bile duct. **A** In the absence of β-glucuronidase producing bacteria, SN38G is excreted without initiating mucositis. **B** Gastrointestinal mucositis is exacerbated through the conversion of SN38G to SN38 by bacterial β-glucuronidase enzymes, exposing intestinal tissue and mucosa to the cytotoxic drug

It is increasingly understood that the gut microbiota can influence every aspect of drug pharmacokinetics through modulating circulating levels, biodistribution, metabolism, and excretion [27], which, by extension, impacts the cytotoxic potential and pharmacodynamics of chemotherapeutics. The capacity for the microbiota to drive drug-induced GI toxicity and GI-M has been best described in the context of irinotecan, a chemotherapeutic drug that undergoes second-pass hepatic recirculation, resulting in its inactive and non-toxic metabolite (SN38G) being excreted into the GIT lumen. Here, bacterial β-glucuronidase (present in a wide array of gut microbes, e.g., *E. coli*) metabolizes and reactivates SN38G into the active cytotoxic metabolite, SN38, triggering mucositis and dose-limiting diarrhea due to direct exposure of SN38 to the GIT mucosa. The severity of irinotecan-induced GI toxicity has been directly linked with host microbiota composition, where in simple terms, GI-M severity is greatest in hosts with more abundant β-glucuronidase producing bacteria capable of converting SN38G to SN38 (Fig. 1) [28]. A wealth of literature from preclinical investigations provides evidence for a suggested causal mechanism, including in germ-free models (mice that are devoid of a microbiota) [29], gnotobiotic models monoassociated with *E. coli* strains with and without genes coding for β-glucuronidase [29], specialized in vitro and ex vivo metabolism and transport studies [30, 31], and through in vivo studies where microbiota are modulated through supplementation with pro- and prebiotics [32–34] or β-glucuronidase inhibitors [35]. In a randomized double-blind clinical trial, Mego et al. highlighted that all grades of diarrhea and GI-M were reduced in patients co-administered a probiotic blend alongside 12 weeks of irinotecan therapy [36], while Kehrer et al. demonstrated that irinotecan-induced diarrhea can be alleviated by depleting the activity of β-glucuronidase within the microbiota through co-administration with the antibiotic, neomycin [32]; thus, confirming that the severity of GI toxicity and GI-M is linked with microbiota composition. However, despite these recent studies solidifying this link between gut microbiota and GI-M severity, establishing a direct causal effect will require further in-depth investigations.

Beyond irinotecan, the microbiota has also been shown to modulate GI-M triggered by 5-fluorouracil (5-FU) [37], methotrexate (MTX) [38], and small molecule tyrosine kinase inhibitors [39]. However, unlike irinotecan, where a depleted gut microbiota leads to reduced GI-M severity, the initiation of GI-M by other chemotherapeutics is typically caused by drug-induced microbiota dysbiosis, with GI-M severity heightened in hosts with depleted microbiota diversity [40]. In this context, the metabolome plays an integral role in GI-M initiation, where reduced SCFA abundance leads to changes in mucosal barrier composition and increases in intestinal permeability, epithelial damage, inflammation, and oxidative stress [41]. Furthermore, the SCFA butyrate has been shown to upregulate Abbc1 in intestinal organoids [42], suggesting that the gut microbiota may influence mucositis initiation by controlling the rate at which enterocytes clear chemotherapy drugs. Importantly, supplementing butyrate and butyrate-producing bacteria during chemotherapy have been shown to be successful interventions for protecting and shielding the microbiota and its metabolome from chemo-induced damage, thus limiting the severity of GI-M [37, 43].

Further studies have provided evidence that an individual’s unique microbiota composition (microbial “fingerprint”) can drastically alter the toxicity profiles of chemotherapeutics through direct drug metabolism by bacterial microbes. For example, 5-FU is directly metabolized by microbes within Firmicutes and Proteobacteria phyla to its inactive metabolite dihydrofluorouracil, which mimics the major host mechanism for drug clearance. By modulating the presence of a key operon necessary for 5-FU inactivation (i.e., preTA operon) within the microbiota, Spanogiannopoulos et al. demonstrated that the bioavailability and efficacy of 5-FU was inhibited in mice colonized with *E. coli* that coded for the preTA operon [44]. It was further demonstrated that marked inter-individual and temporal differences in preTA abundance exist within colorectal cancer patients, suggesting that an individual’s microbial fingerprint will dictate their response to treatment. While toxicity and GI-M were not quantified or monitored in this study, it is expected that this direct drug metabolism by the microbiota alters the relative concentration and exposure of the active cytotoxic drug to intestinal tissue and mucosa.

In addition to influencing drug pharmacokinetics (i.e., absorption, distribution, metabolism, and excretion), an individual’s unique microbiota composition also has the capacity to control mucosal physiology and immunology, thus influencing the sensitivity of the mucosa to injury. This sensitivity is largely due to the microbiota’s ability to control the host’s immune system, with findings from the Human Functional Genomics Project indicating the microbiota is the most influencing factor shaping both resting and stimulated immune responses [45]. Indeed, this concept has been best described in the setting of immunotherapy-induced colitis, which draws some parallels with GI-M. For example, a microbiota enriched for the *Faecalibacterium* genus and other *Firmicute* phyla conferred a more favorable anti-tumor response in patients with melanoma (increased progression-free and overall survival), but also increased the risk of colitis [46]. As a result, strategies to prime the microbiota to optimize immunotherapy responses are being investigated for their safety and efficacy. For a comprehensive review of this topic, please see Zhou et al., 2022 [47].

When it comes to traditionally defined mucositis (i.e., mucosal damage induced by cytotoxic chemotherapy or radiotherapy), the evidence for microbial fingerprints is scarce. However, emerging evidence from preclinical and pilot studies suggests similar findings. For example, it is already known that antibiotics in the lead-up to cancer therapy increases the risk of OM and GI-M [48], suggesting that a disrupted microbiota influences the initiation of mucositis. These findings have been confirmed in a preclinical model of chemotherapy-induced GI-M, in which antibiotic-induced *disruption* of the gut microbiota increased the duration of GI-M [49]. Importantly, this detrimental effect was reversed by fecal microbiota transplantation (FMT), again reiterating that *pre-therapy* microbiota composition influences the initiation of mucositis [49].

In contrast, antibiotic-induced *depletion* of gut microbiota before head and neck radiotherapy has been shown to reduce the severity of OM in rats [50]. Antibiotic use partially explained the loss of microbial diversity in stool samples from allogenic hematopoietic stem cell transplant (allo-HCT) patients receiving preparative conditioning regimens [51]. However, patients not exposed to antibiotics also showed loss of diversity following conditioning regimens, suggesting antibiotics may contribute to some of the diversity loss. Still, host responses to conditioning regimens are likely also contributing to microbial disturbances, with higher intensity regimens resulting in the greatest loss of diversity and the highest proportion of patients with liquid stool consistency (diarrhea) [51]. These contrasting results likely reflect the difference between disruption and depletion or the contextualized differences in the models (i.e., radiation vs chemotherapy, oral vs gut). Nonetheless, these findings clearly implicate the gut microbiota in the initiation of mucositis.

Clinical data exploring this concept is limited; however, a handful of studies support a role for the microbiota in mucositis initiation. A recent review by Fernandez Forne et al., highlights that some studies observed an association between reduced oropharyngeal and oral cavity microbial diversity at baseline and the development of more severe OM during radiation treatment [52]. Al-Qadami and colleagues showed that baseline gut microbiota composition can predict OM severity in patients receiving radiotherapy for head and neck cancer (HNC) [32]. Specifically, in a small cohort of 17 HNC patients, mild (grades 1–2) OM was associated with a high abundance of *Bacteroides*, *Parabacteroides*, *Faecalibacterium*, *Ruminococcaceae*, and *Clostridiales*, whereas severe (grades 3–4) OM was associated with *Bacteroides*, *Faecalibacterium*, *Ruminococcaceae*, *Prevotella*, and *Lachnospiraceae* [32]. Significant increases in the relative abundances of *Eubacterium*, *Victivallis*, and *Ruminococcus* were shown in severe OM compared with mild OM [32].

Further, no tumor recurrence was shown to be associated with the significantly higher relative abundance of *Faecalibacterium*, *Prevotella*, and *Phascolarctobacterium* [32]. However, the causal correlation between dysbiosis and mucositis development has not been definitively proven in clinical settings. Bruno et al. evaluated that the *Porphyromonas* abundance at the baseline of HSCT treatment correlates with the severity of OM. In contrast, there is no difference in the oral mucosa diversity between patients who do or do not develop oral mucositis [53]. Laheij et al. found no association between the diversity of the oral microbiome and the presence (or absence) of oral chronic graft versus host disease (GvHD), with no differences observed between samples collected prior to stem cell conditioning treatment and those collected after [54]. However, the same study did show that an increased abundance of “disease-associated” microbiota was associated with OM, and “caries-associated” microbiota were associated with the absence of OM [54]. In another study by Laheij et al., baseline levels of Streptococcus and Actinomyces (generally associated with oral health) were shown to be associated with an intact oral mucosa following autologous SCT pre-treatment [55]. Covington et al. (2012) also showed that baseline gut microbial profiles of patients prior to receiving pelvic radiotherapy differed between patients who went on to have low toxicity and those with high toxicity, suggesting baseline gut microbiota could be used to predict radiation enteritis [56], with these findings supported by another small study by Wang et al. (2015), also demonstrating a dysbiotic shift of reduced diversity in gut microbial populations prior to pelvic radiotherapy being predictive of diarrhea [57]. A limitation of these studies is the small sample sizes (*n* = 23 and 11, respectively), meaning that no causal link can be established between the etiology of radiotherapy-induced diarrhea and any of the bacterial taxa investigated. However, further evidence of gut microbiota changes caused by radiotherapy is provided in a recent systematic review by Wang et al. (2021), with key findings of pelvic radiotherapy (and its accompanying gut damage) being associated with disruption of gut microbiota, with diversity decreased before, during, and after radiotherapy [58].

### Phase II/III: Messaging, signaling, and amplification

Initiation leads to messaging and signaling, and this then leads to the amplification stage of mucositis through a positive feedback loop with IL-1β, IL-6, and TNF-α being further transcribed [4, 59, 60], expediting and sustaining tissue damage of the mucosal cells lining the alimentary tract. This cascade of inflammatory pathways also includes NF*κ*B and the retinoblastoma control protein, p53, which both function in increasing the amplification stage of mucositis [4]. The resulting inflammation leads to ulceration and continues to provide a positive feedback loop, increasing transcription of pro-inflammatory cytokines, continuing to amplifying the tissue damage [4].

As already described by many in the literature, both oral and gut microbiota undergo significant changes following cancer treatments [37, 40, 55, 61–75]. While this concept is not debated, what has remained unclear is whether these changes are simply a *consequence* of treatment (and likely mucositis itself) or if they *causally contribute* to the development, progression and, ultimately, clinical impact of mucositis. Dissecting cause and consequence in this setting has been extremely challenging due to the highly dynamic nature of mucositis and microbial changes. However, evidence continues to support the concept of a causal role largely mediated through bacterial by-products, such as lipopolysaccharide (LPS) [76]. LPS is produced by Gram-negative pathogens and is a well-known ligand for the pattern recognition receptor, TLR4. Of note, TLR4 is expressed on the basolateral surface of enterocytes and is only activated if LPS can translocate across the mucosal barrier, i.e., in the context of mucositis [76, 77]. When activated, TLR4 initiates an intensive and rapid inflammatory response to eradicate the invading pathogen [76]. Certainly, TLR4 and LPS levels are increased after chemotherapy [77, 78], suggesting microbial-mediated tissue injury and inflammatory responses. Of interest, Wardill and colleagues showed that TLR4 knockout BALB/c mice were less likely to develop severe mucositis and diarrhea after irinotecan treatment [77, 79], possibly due to the absence of an IL-6 response [80]. However, it is important to note that contradictory findings have been observed, with TLR4 knockout C57BL6 mice more susceptible to mucositis induced by the same chemotherapeutic agent, irinotecan [81]. What remains unclear from each of these studies is whether TLR4 expressed on epithelial, immune of other cell populations are responsible for the findings, and given the differences in immune profiles in BALB/c vs C57BL6 mice, this may be an explanation for these contradictory findings [82]. Of interest, in the context of radiotherapy, TLR4 deficiency appears to increase the risk of GI-M. However, TLR4 knockout mice had a less severe inflammatory response compared to their wild-type counterparts [83]. These findings suggests that a finely tuned balance of TLR4 is needed to maintain mucosal homeostasis, and both a pathological upregulation or complete depletion of TLR4 is detrimental.

TLR4 is less abundant in the oral cavity, but is nonetheless involved in maintaining tissue homeostasis. A compensatory internalization of TLR4 minimizes LPS-induced activation and, thus, mucosal inflammation. In the context of OM, it appears this compensation is maintained, with oral expression of TLR4 and its accessory protein MyD88 decreased after MTX, limiting their stimulation and subsequent activation [84]. However, whether this is truly protective or indeed impairs mucosal recovery via protective immune responses against pathogens is unclear, and as such, the causal role of this mechanism in OM is unclear.

Unlike TLR4, TLR2 detects and responds to a variety of microbial products generally produced by Gram-*positive* microbes, including lipoproteins, peptidoglycans and lipoteichoic acid. Frank and colleagues [85] showed that genetic deletion of TLR2 in mice exacerbated GI-M caused by the chemotherapy drug, MTX [85]. Although TLR2 is also highly expressed in the basal layer of the gingiva and plays an important role in tissue homeostasis, its role in OM has not been explored. In addition to TLR2 and 4, other TLRs have also been explored in the context of mucositis, largely TLR5 and 9, although results are sporadic and somewhat heterogeneous. For example, the only study to investigate TLR5 showed that CBLB502 (TLR5 agonist) protected the oral mucosa from radiation-induced damage, also preventing associated weight loss [86, 87]. Alternatively, antagonizing TLR9 can reduce intestinal injury caused by some chemotherapeutic agents, and complete genetic deletion has been shown to preserve intestinal architecture after irinotecan chemotherapy [88]. Cumulatively, these data show that interactions between the microbiota and the host profoundly influence the pathogenesis of mucositis and thus suggest a causal role for the microbiota’s involvement.

In addition to pathological signaling via TLRs, the microbiota interacts with its host via the production of beneficial by-products, namely SCFAs. SCFAs inhibit NF*κ*B activation [20, 89], stimulate mucosal repair, reinforce tight junction assembly/stability [22], stimulate mucus production, and recruit regulatory T cells, which minimize damaging inflammation [20]. The role of SCFA in reinforcing tight junctions has been demonstrated in both cells [22] and mice [90], showing that pre-treatment of cells with SCFAs acetate, propionate, and butyrate prior to administration of 5-FU to cells, and pre-treatment of mice with butyrate prior to gemcitabine, results in increased expression of occludin (tight junction protein) and increased MUC2 expression in cells [22], and increased goblet cell counts in butyrate-treated mice compared to gemcitabine treated mice [90]. Similarly, findings have been shown in organoids, where administration of butyrate alongside methotrexate reduced cellular damage and inflammation. Several other studies also highlight the beneficial effects of postbiotics, each describing slightly different mechanisms of protection [22, 43, 90, 91]. Together, these findings suggest that SCFAs prevent chemotherapy-induced damage by maintaining tight junction integrity and promoting mucus production, but again, causation is challenging to define [22, 90].

Diet composition also impacts the gut microbiota and its metabolic output. In a study by Gallotti et al., a high-fiber diet (containing 15% pectin) ameliorated chemotherapy-induced damage in mice, by preventing villus shortening in the small intestine and reducing leukocyte infiltration [92]. The high-fiber diet also protected epithelial barrier integrity and reduced permeability [92]. Interestingly, oral administration of acetate did not have the same effects [92], potentially due to pectin having prebiotic action to stimulate the production of SCFA-producing microbiota, whereas acetate is absorbed and metabolized quickly and is unable to modulate the microbiota when administered this way [92]. Pectin also blocks TLR2-dependent inflammation and can prevent mucositis through this mechanism [93]. Despite microbiota not being investigated in this study, the TLR2-driven mechanisms (and their inhibition) may be potentially stemming from upstream microbial modulation resulting from pectin.

In addition to diet, the microbiota can be augmented through the direct provision of microbes either as select strains (i.e., probiotics) or complex ecosystems (i.e., fecal microbiota transplantation (FMT)). Somewhat counterintuitively, probiotics have documented effects for both GI-M and OM, largely due to the gut-oral axis in which the immune system is the systemic “middleman” [67]. In general, probiotics evoke a down-regulation in immune response, improving the mucosal barrier function [73]. In preclinical studies, *L. reuteri* maintained oral epithelial thickness and protected against 5-FU-induced OM in mice [94]. Further, the topical application of *Streptococcus salivarius K12* reduced the size of radiation-induced OM in hamsters and the oral anaerobes composition [95], and in a clinical trial in patients receiving radiotherapy, topical application of *Streptococcus salivarius K12* probiotic lozenges significantly reduced the incidence, time to onset, and duration of OM [96]. The mechanisms of protection from *S. salivarius* K12 remain undefined [97]. The heterogeneous response may be due to differences in pre-treatment microbiota that respond differently to the radiotherapy treatment, potentially non-specific protective responses from *S. salivarius* K12, or maintenance of the microbial environment, and again highlights the inherent difficulties of analyzing a true causal role of microbiota in OM [96, 97].

More importantly, the impact of probiotic interventions on OM has been confirmed through meta-analysis of 708 patients [68]. Of interest, even with a highly heterogeneous collection of studies with varying probiotic formulations and OM assessment tools, a substantive benefit was identified underscoring the likely involvement of the gut microbiota in OM pathogenesis. This very much mirrors the context for GI-M, with the clinical practice guidelines established by the Multinational Association for Supportive Care in Cancer (MASCC) recommending the use of *Lactobacillus* containing probiotics currently recommended for the prevention/management of GI-M in certain settings [3, 69]. However, it is important to note that probiotics are not effective in *all* settings [98]. Whether this is due to differences in the subtle mechanisms leading to mucositis across these settings, or the limitations of the data available is unclear. Nonetheless, direct administration of probiotics has some effect of OM and GI-M, underscoring the likely contribution of the microbiota to mucositis pathogenesis.

A potential reason for the limited efficacy of some probiotics is their narrow diversity and microbial load [99]. In contrast, fecal microbiota transplantation (FMT) achieves a greater microbial load as it is prepared using an entire microbial ecosystem collected from fecal or saliva samples [100]. As such, FMT has been increasing investigated in the context of mucositis [101, 102], with preclinical evidence showing FMT mitigates disruption of the host’s microbiota following chemotherapy and reduces opportunistic and resistant microbe proliferation [100]. Oral microbiota transplantation (OMT) is an underdeveloped therapy; however, a useful tool to investigate the effect of the oral microbiota on OM development and recovery. In one preclinical study, OMT lowered the OM severity by favoring the epithelium and tongue papillae reconstruction post-radiotherapy. In addition, the OMT cohort presented more homogeneous diversity and composition [103].

### Phase IV/V: Ulceration and healing

Messaging, signaling and amplification of inflammatory signals results in the eventual breakdown of the mucosa, presenting as ulceration throughout the entire alimentary tract [4]. As described earlier, the microbiota is almost certainly involved in the mechanisms that dictate mucosal inflammation and injury in mucositis development. As such, it is only natural that these same mechanisms extend into the transition into the ulcerative phase. Indeed, historically, it has been the ulcerative phase where the microbiota has been already implicated, with resident microbes colonizing at the sites of mucosal injury and eventually translocating into the bloodstream. These microbes heavily influence the symptoms of mucositis by affecting fluid movement within the tissue, increasing diarrhea, motility, and nociception, leading to increased pain and prevalence of symptoms [75]. Microbes can also influence bile acid metabolism, increasing toxic secondary bile acids with a high osmotic pull, increasing diarrhea and dehydration [75, 104]. These microbes are toxic to the mucosa, contributing to increased ulceration and clinical manifestations of mucositis [75]. Beyond the mechanisms that lead to ulceration and the highly opportunistic interaction between the mucosa and microbes, there are limited avenues for mechanistic involvement in mucosal ulceration. Where the microbiota really becomes causally relevant is dictating the duration of mucosal injury/ulceration via its influence on mucosal recovery (healing).

As outlined in the 2004 pathobiological model of mucositis, mucosal healing is initiated by extracellular matrix signaling, stimulating cellular proliferation and the subsequent re-establishment of the mucosal barrier [4]. The microbiota exerts profound control of the mucosa’s capacity to recover after injury, for instance, through the production of SCFAs, which directly influence enterocyte proliferation [21, 105]. Although scarcely investigated in the setting of mucositis, the capacity of SCFAs to influence mucosal restitution has been clearly demonstrated in in vitro and in vivo settings with relevance to inflammatory bowel disease (IBD), reviewed in detail by Parada Venegas et al., describing mechanisms by which SCFA have been shown to maintain intestinal homeostasis, potentially contributing to mucosal healing in other settings, such as GI-M. In Caco-2 cells, butyrate promotes tight junction assembly through the redistribution of zona occludens (ZO-1) and occludin, mediated through AMP-activated protein kinase (AMPK) activation and inhibition of myosin light chain kinase (MLCK)/MLC2 and protein kinase C (PKC) β2 [21, 106, 107], and upregulated the IL-10 receptor (IL-10RA) through feed-forward regulation of STAT3 [21, 23], suggesting SCFA-mediated mechanisms of epithelial barrier repair. SCFAs are the main energy source of colonocytes, and thus when deficient (as in mucositis), mucosal recovery is impaired hence identifying a causal role for how the microbiota augments mucositis pathogenesis. Further supporting a role for the microbiota in influencing mucosal healing via the production of SCFAs is translational data and insights gained from the use of FMT, which, when used in the context of graft versus host disease where there is extensive mucosal damage, results in an increase in mucosal integrity and barrier function [108]. In the context of mucositis, the importance of the microbiota to mucosal healing is illustrated by the detrimental effects of antibiotics, which, when given prior to chemotherapy, delay mucosal recovery, thus increasing the duration of ulcerative mucositis and associated symptoms, morbidity and mortality [49]. This influence over healing is likely to be not only related to the direct effect of SCFAs on enterocyte repopulation/proliferation but also on the re-establishment of mucosal barrier integrity (via tight junction assembly) and restitution of immune homeostasis, each of which SCFAs are well known to influence [21, 105, 109].

Mucosal recovery may also be influenced by cell wall components of microorganisms or by other bacterial/fungal products besides SCFAs. For instance, the oral bacterium *Porphyromonas gingivalis* was shown to inhibit wound healing in vitro [110]. Responsible for this effect were the Arg- and Lys-gingipains and the absence of a capsular polysaccharide of certain strains exposing cell wall-bound structures [110]. The major fimbriae and LPS of *P. gingivalis* were not involved in delayed wound healing [110]. Also, *Candida glabrata* and *Candida kefyr* inhibited cell migration in vitro [111]. These two oral microorganisms probably influenced cell migration, proliferation, and metabolism of epithelial cells, but not the reproductive capacity of these cells [112]. Others found that the bacterial load and oral microbiome composition were important factors for wound healing in vitro [113].

Ultimately, via its influence on proliferation, inflammation, and barrier function, the microbiota is critical in dictating the rate of mucosal recovery and, thus, the duration of mucositis. With an increasing body of evidence indicating that the burden of mucositis (i.e., depth and duration of mucosal injury) dictates the severity of secondary symptoms and complications (e.g., infection), the microbiota’s clear role in regulating the healing phase is of considerable interest. Further to this, there are a few challenges that make augmenting the microbiota during peak mucositis exceptionally challenging and high risk (e.g., infection); as such, targeting the microbiota to accelerate healing may offer a more feasible method for controlling mucositis and its associated complications. [109].

## Summary of the microbiota’s causal role in mucositis

Emerging fundamental evidence suggests that the microbiota causally contributes to the pathogenesis of mucositis, although studies that are designed to strictly assess causality remain scarce and methodologically challenging. The ability of various microbial manipulative strategies to alter mucositis development/severity, while not strictly causative, certainly add strength to the growing evidence that microbial-mucosal-immune interactions serve to exacerbate or perpetuate cytotoxic therapy-related mucosal injury. However, what remains unclear is the specific mechanisms by which microbial interventions exert their benefit and, thus, those that outline precisely how key microbial taxa contribute to mucositis pathogenesis [114, 115]. Several mechanistic observations have been made from studies testing various microbial interventions, including changes in drug pharmacokinetics, enhanced luminal acidification, mucus production, tight junction integrity, reduced inflammation, and normalized bile acid profiles [20]. Ultimately, it is highly unlikely that a single mechanism can be identified. Instead, it is most plausible that multiple events involving the microbiota, mucosa, and immune system operate in concert to dictate the homeostatic milieu of the local and system environment, thus, the severity of mucositis and its associated symptoms. It is critical to acknowledge the ineffective results of certain microbial-based interventions in controlling mucositis [98], with lack of efficacy reflecting the heterogeneity between studies regarding drug regimens and radiotherapy modalities, mucositis measures and pathophysiology assessment [98]. As such, for meaningful advances in understanding how to modulate the microbiota to control mucositis effectively, the field must adopt consistent methodological approaches concerning microbiome analyses, intervention design and mucositis assessment.

**Fig. 2:**
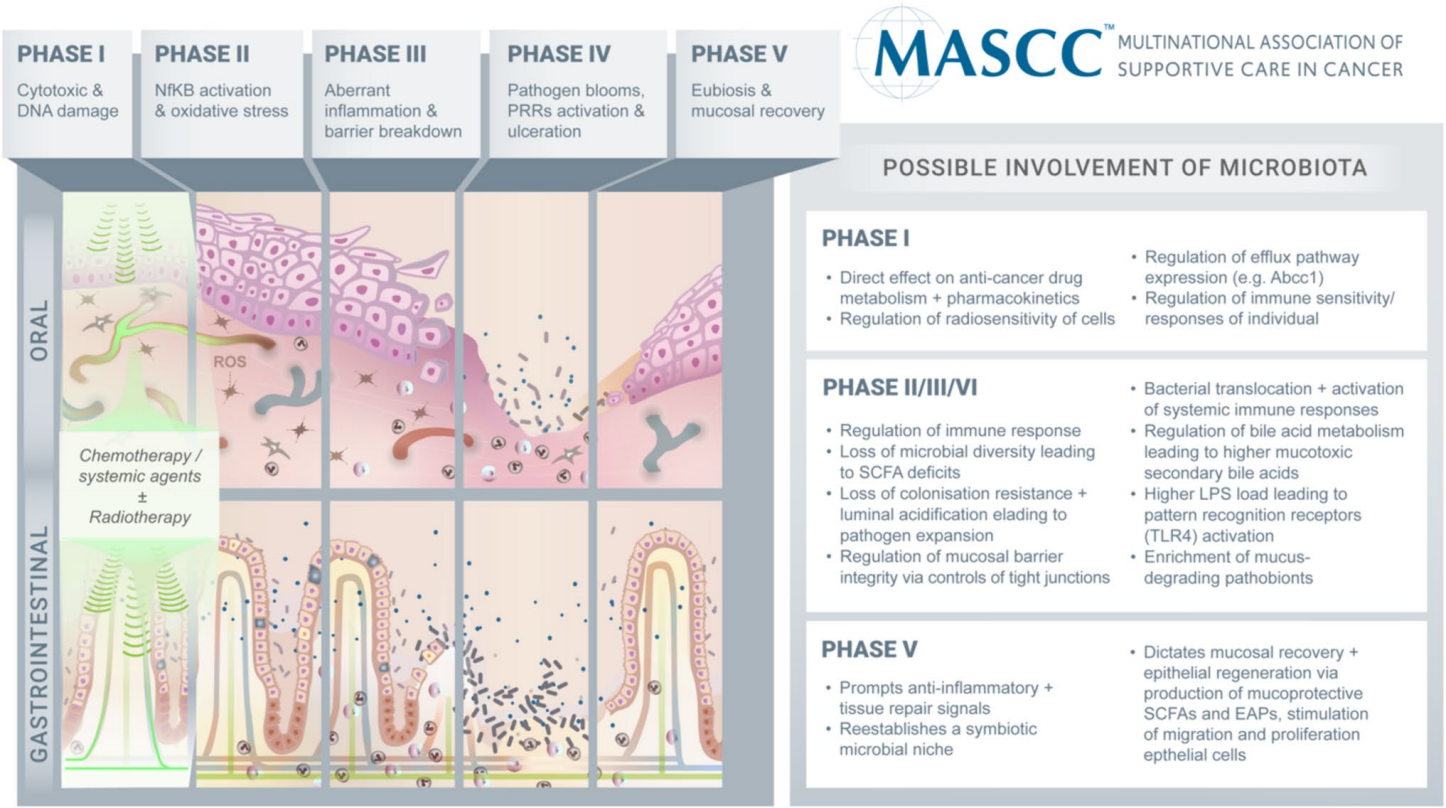
**5-**phase pathogenesis of mucositis and involvement of the microbiota

While we have outlined a range of studies that suggest a causal role for the microbiota throughout all stages of mucositis development (summarized in Table 1 and Fig. 1), we must acknowledge that we have only provided a snapshot of the data available on this topic and have likely been influenced by publication biases that prioritize and overinflate positive findings and thus may over-exaggerate the extrapolation we have made in connecting positive interventional studies with mechanistic possibilities. Furthermore, the way microorganisms live within the microbiome ecosystem is extremely complex. With the current techniques, especially those that appear to dominate the mucositis literature, we can only study an overly simplified version of this coexistence. It is critical that moving forward, we appreciate this complexity with greater appreciation and design studies with paralleled ex vivo approaches that aim to dissect the causal regulation of mucositis via microbial signaling. In doing so, it is also important that the entire microbiota be truly appreciated, with our siloed vision on just the bacteriome being challenged to include the archaea domain, fungi and protozoa from the eukaryote domain, and viruses (including bacteriophages). In parallel, efforts to capture functionally relevant—outs are critical, and attempts to move beyond solely performing 16S rRNA gene sequencing should be prioritized. This may include the addition of SCFA analyses or more sophisticated genomic sequencing that provides higher-resolution detail on the functionality of microbial taxa and specific strains. This will undoubtedly provide the insight required to drive more substantive advances in our understanding of the microbiota and its influence on mucositis. Of course, this must also be performed longitudinally to appreciate the dynamic changes in the microbiota and how they relate to key milestones in mucosal injury and associated symptomology (Fig. 2).

**Table 1:** Potential roles of microbiota through stages of mucositis pathogenesis

Pathogenesis stage	Phase I	Phase II–IV	Phase V
Microbial contribution	• Drug metabolism • Drug efflux pathways • Baseline immune function	• Exacerbated pro-inflammatory immune responses • Mucus degradation • Perturbed tight junctions and barrier function • Bile acid metabolism	• Epithelial proliferation and migration • Tight junction re-assembly • Anti-inflammatory responses
Novel questions and opportunities for investigation	• Can the microbiota be exploited/augmented to slow mucosal proliferation and decrease sensitivity to cytotoxic injury? • Can the microbiota be augmented to enhance local drug efflux mechanisms to minimize mucositis? • What is the ideal microbial fingerprint and how is it achieved before starting therapy? • How can we identify microbial fingerprints rapidly? • How can the microbiota be augmented to minimize mucositis without impairing tumor kill?	• What drives microbial injury in mucositis? Is it the chicken or the egg? • What is the best way of achieving microbial stability given the hostility of the alimentary tract during mucositis and confounding factors (e.g., antibiotics, dietary changes)? • How is the oral microbiota best supported?	• Are microbes or microbial compounds (SCFAs) most effective in accelerating healing?

Finally, it is critical that we cooperate to advance our understanding of microbe-mucositis interactions and adopt consistent approaches in project methodology. Although many studies implicate the microbiome in mucositis development, unfortunately, the field has struggled to progress beyond this hype and deliver translationally meaningful insights. In fact, there remains only one clinical practice guideline recommending a microbial-based intervention for mucositis, with *Lactobacillus* containing probiotics only recommended in a relatively restricted patient population [116]. This reflects the variable nature of microbiome science and, thus, the inconsistency in methodological approaches employed across studies, making comparisons across studies exceptionally difficult. In addition to more consistent mucositis assessment, emphasis should be placed on adequate reporting of studies to ensure reproducibility with respect to intervention design/delivery, sample collection, processing and sequencing. Numerous tools exist to guide people in these areas to ensure adequate reporting; thus, replication can be achieved [117, 118].

### Methods of sample collection and data analysis

The oral cavity consists of several microbial niches, such as the gingiva, tongue, dental tissue, and palate. However, significant cross-niche correlations exist between different sample types [119] and need to be considered relative to the research question. Most large studies into the oral microbiome use saliva or an oral rinse, as they are easy to collect [120]. While these sample types do not represent a specific oral niche, they do resemble the microbiota on mucosal surfaces [119, 121]. While access to the oral cavity is relatively simple, the rest of the gastrointestinal tract is not well accessible, leading to a more clinical focus of oral studies and animal focus for specific regions of the gastrointestinal tract. In animal models studying the gut microbiome in relation to gastrointestinal mucositis, the possibilities for sampling a specific part of the intestine are greater than in humans. Hence, the ability to sample the caecal and colon content in euthanized rats and mice [50, 122, 123], and feces in live mice [124, 125] are reported, while in humans, feces are sampled rather than caecal or colon content [125, 126]. One distinct advantage of feces is the ability to conduct repeated sampling.

Once samples are collected, careful consideration must be taken in DNA extraction and library preparation. It is critically important to reduce variability in both methods and reagents used for these processes and to include a blank sample at every step to detect possible contamination [127]. Among the sequencing methods, there is the possibility of sequencing a specific gene (16S rRNA gene for bacteria) or the whole bacterial genome [128, 129]. The method should again be chosen depending on the hypothesis/goal of the study. To find the phylogenetic and microbial community information of the sample, sequencing the 16S rRNA is a fast and cost-effective method [129–131]. Another advantage of target-gene sequencing is that the presence of human DNA in the sample has little or no effect on the results [128]. Whole genome sequencing, also called metagenomics analysis, extracts more genomic information and enables us to find a higher taxonomic resolution [128, 129, 132].

In contrast to the greater amount of data, it is more expensive and requires more careful analysis. A great advantage is the reduction of biases caused by the library construction steps and database choice, as the results are inferred directly from the genomics of the sample [128]. It is beyond the scope of this review to suggest how microbiome datasets should be analyzed, as there are many thorough resources available in this area. However, understanding the points of confusion and standardizing the possible methodological and clinical points is important to achieve clinically impactful results [128, 129].

## Conclusions and future perspectives

It has long been known that mucositis is *associated* with microbial changes, yet the *causal* basis for these findings has been challenging to confirm. As microbiome science evolves, our understanding of the causal basis for the microbiota’s involvement in mucositis will continue to grow in depth and complexity. Ultimately, the mechanistic basis for the microbiota in mucositis is best explored using germ-free models, which can be selectively colonized with specific microbes or microbial communities. In addition, it is likely that the effect(s) of certain microbial-based therapeutics will also continue to strengthen our understanding of the microbiota’s involvement in mucositis development. However, it is critical that we continue to approach and interpret these studies appropriately and acknowledge the technical limitations of current approaches as well as the numerous confounding factors that may render microbial interventions ineffective. Ultimately, the end goal in understanding the causal basis for the microbiota in mucositis development should be to design and implement microbial interventions that improve mucositis burden. As such, this ethos must remain front and center when designing these studies. Emphasizing clinically meaningful endpoints (e.g., patient-reported outcome measures) while also collecting appropriate biospecimens to conduct translational and mechanistic studies will create the knowledge base that is needed to advance mucositis prevention and management while also continuing to advance the pathobiological model to reflect the modern understanding of this highly prevalent and burdensome complication of cancer therapy.

## Data Availability

No datasets were generated or analysed during the current study.
